# Clemastine Inhibits the Biofilm and Hemolytic of Staphylococcus aureus through the GdpP Protein

**DOI:** 10.1128/spectrum.00541-21

**Published:** 2022-03-02

**Authors:** Yongpeng Shang, Jie Guo, Yuxi Zhao, Junwen Chen, Qingyin Meng, Di Qu, Jinxin Zheng, Zhijian Yu, Yang Wu, Qiwen Deng

**Affiliations:** a Department of Infectious Diseases and the Key Lab of Endogenous Infection, Shenzhen Nanshan People's Hospital, the 6th Affiliated Hospital of Shenzhen University Health Science Center, Shenzhen, China; b National-Regional Key Technology Engineering Laboratory for Medical Ultrasound, Guangdong Key Laboratory for Biomedical Measurements and Ultrasound Imaging, School of Medicine, Shenzhen University, Shenzhen, China; c Key Laboratory of Medical Molecular Virology of Ministries of Education and Health, School of Basic Medical Science and Institutes of Biomedical Sciences, Shanghai Medical College of Fudan University, Shanghai, China; Peking University People's Hospital

**Keywords:** biofilm, clemastine, GdpP, hemolysis, proteomic, *Staphylococcus aureus*

## Abstract

Staphylococcus aureus poses a significant threat to human health due to its virulence and multidrug resistance. In addition, recalcitrant biofilm formation of S. aureus often results in chronic infection and the treatment tolerance toward the traditional antibiotics. Thus, the development of novel antimicrobial agents capable to inhibit or eradicate S. aureus biofilm formation does matter. Here, we demonstrated that clemastine showed slight bacteriostatic activity and enhanced the antibacterial activity of oxacillin against S. aureus. Moreover, the dramatic inhibition of biofilm formation was found in clinical S. aureus strains by clemastine. Clemastine inhibited the release of eDNA during the biofilm formation and decreased the S. aureus hemolytic activity. Moreover, the S. aureus SA113 treated with clemastine displayed the decreased transcriptional level of the biofilm formation relevant genes (*fnbB*, *icaA*, and *icaB*), virulence genes (*hlg, hld*, *lukde, lukpvl*, *beta-PSM*, *delta-PSM,* and *cap5A*), and the regulatory genes *agrA*. The proteomics analysis of SA113 treated with clemastine demonstrated the significant changes in levels of biofilm-related proteins (stress response regulators ClpB and GroS, ATP-binding proteins, and urease metabolism), virulence-related proteins (SspA, superantigen, and VWbp), and methicillin resistance-related proteins (glutamine metabolism). The genetic mutations on *gdpP* (cyclic di-AMP phosphodiesterase) were found in the clemastine-induced tolerant derivative isolate by whole-genome sequencing. Furthermore, the interaction between clemastine and GdpP protein was demonstrated by the molecular docking, *gdpP* overexpression experiment, and thermal stability assay. Conclusively, clemastine might exert its inhibitory effects against the biofilm formation and hemolysis in S. aureus through targeting GdpP protein.

**IMPORTANCE** The biofilm formation, which protects bacteria from stresses, including antibiotics and host immune responses, can be commonly found in clinical S. aureus isolates worldwide. Treatment failure of traditional antibiotics in biofilm-associated S. aureus infections remains a serious challenge. The novel anti-biofilm drug is urgently needed to address the looming crisis. In this study, clemastine, which is a histamine receptor H1 (HRH1) antagonist, was found to have a novel role of the significant inhibition against the biofilm formation and hemolytic activity of S. aureus and enhanced antibacterial activity against S. aureus when used in combination with oxacillin by targeting the GdpP protein. The discovery of this study identified novel use and mechanism of action of clemastine as a potential anti-biofilm drug for clinical application for S. aureus infectious.

## INTRODUCTION

As one of the most frequent human pathogens, Staphylococcus aureus can be isolated from a wide spectrum of clinical samples and is often commonly found in the commensal status in the nasopharynx, skin, and some different organs of the healthy human population. S. aureus infection results in a variety of infectious diseases, such as skin and soft tissue infection, pneumonia, endocarditis, and bacteremia (1, 2). Moreover, methicillin-resistant Staphylococcus aureus (MRSA) has been considered one of the most notorious multidrug-resistant bacteria, and the hospital-acquired infection caused by MRSA is significantly associated with high morbidity in clinics. Recently, multidrug-resistant S. aureus nonsusceptible to the last-resort antibiotics, including vancomycin, linezolid, and daptomycin, has been increasingly reported. This phenomenon has limited the available choice of antibiotics for the treatment of S. aureus infection (2, 3). Moreover, most clinical S. aureus isolates can promote biofilm formation, which has become an additional challenge for the effective treatment of S. aureus infection. Biofilm-related bacteria can often secrete a self-encasing extracellular matrix, which decreases their susceptibility toward a wide spectrum of antibiotic therapy and protects them from host immune elimination (4). Therefore, the development of the novel antimicrobial agents as the available treatment choice is urgent for clinicians to improve the prognosis of multidrug-resistant and biofilm-associated S. aureus infection.

The developmental stages of biofilm formation can be divided into four major steps: initial attachment, irreversible attachment, maturation, and dispersion (5). During the initial attachment, the individual planktonic cells of S. aureus could adhere to the inert or biotic surfaces and gradually tighten the attachment of the “new surface” by constantly secreting and generating the anchored proteins such as FnBP, Clf, and SasG (6, 7). The intercellular aggregation of bacterial cells can implicate the symbol of S. aureus biofilm maturation and often depends on the production of the biofilm matrix, which is composed of polysaccharide intercellular adhesion (PIA) or poly-N-acetylglucosamine (PNAG), extracellular DNA (eDNA), and several functional proteins (Aur, Bap, and CcpA). The biofilm maturation often requires the participation of PIA which is encoded by the *ica* gene locus, upregulated by SarA and SigB, and downregulated by LuxS (8, 9). Moreover, biofilm maturation often involves the participation of extracellular eDNA and functional proteins in a PIA-independent manner (10). The biofilm-associated eDNA level is associated with the intracellular concentration of cyclic di-AMP (cyclic di-AMP) phosphodiesterase (*gdpP*), which participates in the degradation of the second messenger cAMP, and the transcription factors *xdrA* and *cidA*, which facilitate the translation of holins responsible for autolysis (10, 11). During the process of biofilm dispersion, the single cells or large bacteria clusters are intermittently released from the biofilm-associated chronic infections, such as endocarditis and implant-related infections (5). Autolysis and extracellular protease involved in SarA and Agr quorum-sensing system participate in the control of biofilm dispersal activity (12, 13).

Following the successful colonization of S. aureus on the infected tissues of hosts, the pathogenicity of S. aureus can partly be explained by the release of a variety of toxins and virulence factors, including hemolysins, the Panton-Valentine leucocidin (PVL), phenol soluble modulin (PSM), staphopain B, and capsule. The toxins and virulence factors synthesized by S. aureus can also directly interact with host cells and modulate the host immune response (14). Notably, several S. aureus virulence factors, including α- and γ-hemolysin, PVL, and PSM, belong to the family of pore-forming toxins (PFTs) that can form aqueous channels in host cells and activate inflammasome for the induction of necrotic cell death. Of these, the hemolysins have been considered one of the most important virulence factors of S. aureus strains and contribute to the development of host inflammation and disease progression (15).

To inhibit the biofilm formation of S. aureus and suppress the tissue damage caused by the toxins and virulence factors, new antibacterial drugs are urgently needed in clinics. Screening libraries have been widely used and demonstrated as an effective way to discover new drugs for the inhibition of biofilm formation and virulence of S. aureus (16). Clemastine is a histamine receptor H1 (HRH1) antagonist and has been approved for clinical use by the FDA for over 20 years. Clemastine has indicated a favorable safety profile and has been widely used for alleviating the symptoms of allergic rhinitis, the common cold, and allergic urticaria (17). In this study, the inhibitory effect of clemastine on the planktonic growth and biofilm formation of S. aureus has been demonstrated. Moreover, clemastine exhibited slight antibacterial activity against Enterococcus faecalis, Streptococcus agalactiae, Acinetobacter baumannii, and Escherichia coli. Furthermore, clemastine could block the hemolytic activity of S. aureus. After *in vitro* exposure to clemastine for 30 days, clemastine-induced tolerant S. aureus lost its capacity for biofilm formation, which might be explained by the genetic mutation of *gdpP* under the clemastine pressure. Molecular docking analysis and the overexpression experiments further demonstrated that *gdpP* might be the target site of clemastine in S. aureus. This study offers a new potential application for clemastine in the treatment of bacterial infections.

## RESULTS

### The slight inhibition of the planktonic growth of S. aureus by clemastine.

**FIG 1:**
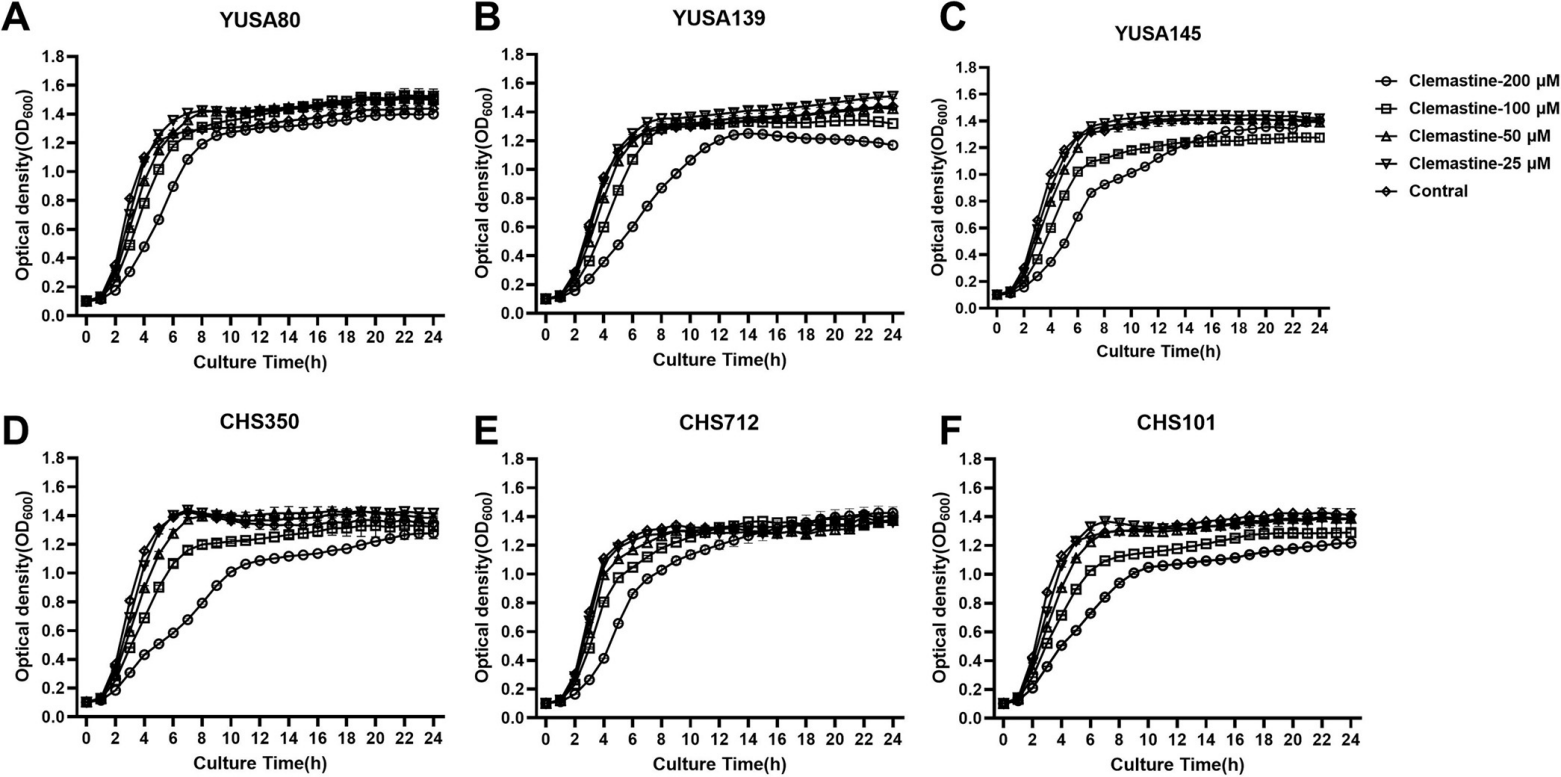
The inhibition of the planktonic growth of S. aureus by clemastine. Liquid cultures of the (A-F) YuSA80, YUSA139, YuSA145, CHS350, CHS712, and CHS101 strains after overnight incubation for 12 h were diluted 1:200 into tryptic soy broth (TSB) containing clemastine (25, 50, 100, and 200 μM). Then, the bacteria dilution was grown at 37°C with shaking at 220 rpm and growth curves were monitored by measuring the optical density at 600 nm (OD_600_) at indicated time points until 24 h in the bacteria automatic growth curve instrument. The experiments were repeated three times, and error bars indicate the standard deviation. The 200 μM clemastine did not affect the planktonic growth of six S. aureus until 24 h.

The impact of clemastine on bacteria planktonic growth were investigated by a series of concentration (25, 50, 100, and 200 μM) in clinical isolates of S. aureus YuSA80, YUSA139, YuSA145, CHS350, CHS712, and CHS101, indicating that clemastine with the concentration of ≥200 μM could slightly and transiently inhibit the planktonic growth from the initial phase after drug exposure and that the S. aureus planktonic growth would be almost restored the growth capacity after 24 h of drug exposure (Fig. 1). In addition, after exposure to the clemastine with a concentration of ≥100 μM, the planktonic growth of E. faecalis, S. agalactiae, E. coli, and A. baumannii would be slightly decreased and in this study, no inhibitory effect of clemastine was found in Klebsiella pneumoniae (Fig. S1). Furthermore, the synergistic effect of clemastine (50 μM) combined with oxacillin, vancomycin, or linezolid on the planktonic growth of S. aureus were assayed by Bioscreen C automatic growth curve analyzer, suggesting clemastine could enhance the antibacterial activity when combined with the subinhibitory concentration of oxacillin (32 μg/mL, 8 μg/mL, or 0.5 μg/mL) in MRSA CHS350, Mu50, CHS691 and MSSA SA113 (Fig. S2A to D). No synergistic effect of clemastine (50 μM) with linezolid and vancomycin was found in four S. aureus isolates by the bacteria growth curve (Fig. S2).

### Influence of clemastine on the biofilm formation and hemolytic activity of S. aureus.

**FIG 2:**
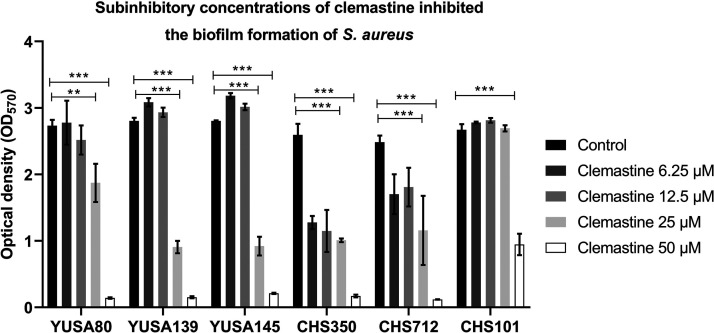
The clemastine with a series of inhibitory concentrations inhibited the biofilm formation of S. aureus by the crystal violet staining method. YuSA80, YUSA139, YuSA145, CHS350, CHS712, and CHS101 strains were incubated with clemastine 24 h at a series of concentrations of 0, 6.25, 12.5, 25, and 50 μM. The biofilm was then measured by crystal violet staining. The biofilm formation of six S. aureus strains was inhibited by clemastine at 50 μM. The data presented was the average of three independent experiments (mean ± SD). Compared with control, *, *P* < 0.05; **, *P* < 0.01; ***, *P* < 0.001; (independent sample *t* test).

**FIG 3:**
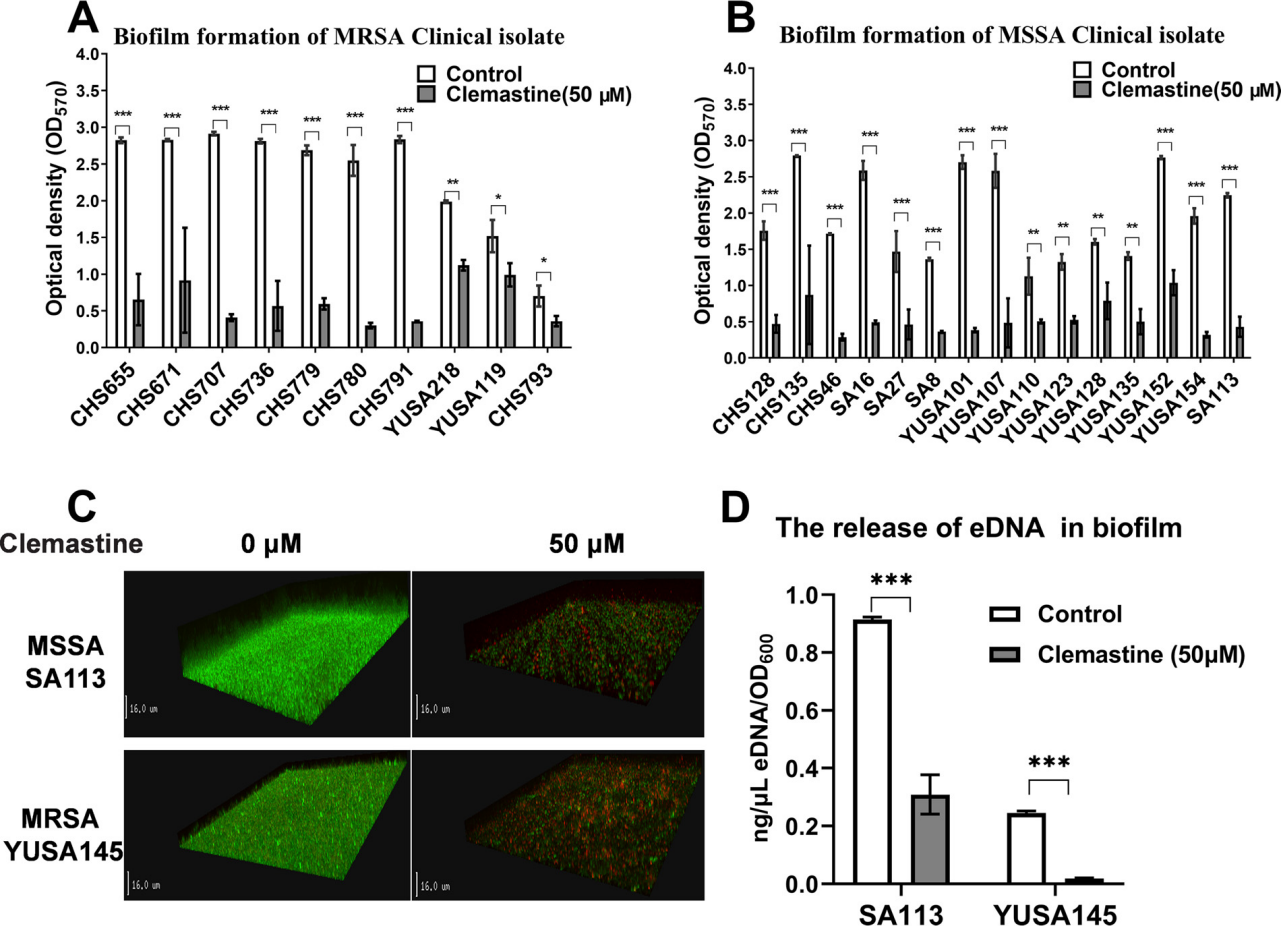
The clemastine at 50 μM inhibited the biofilm formation of S. aureus clinical isolates and decreased the release of eDNA from S. aureus biofilm. Inhibition of the biofilm formation of clinical isolates of 10 MRSA (A) and 15 MSSA (B) were inhibited by clemastine at 50 μM for 24 h by crystal violet staining. (C) The clemastine inhibited the biofilm formation of S. aureus observed by CLSM. Twenty-four-hour-old biofilms of MSSA SA113 and MRSA YUSA145 were grown on cover glass in a cell culture dish and observed by CLSM. Three-dimensional (3D) structural images were reconstructed. Viable and dead cells were stained green (SYTO9) and red (PI), respectively. (D) Clemastine inhibited the release of eDNA in the biofilm of the SA113 and YUSA145 strains. The release of eDNA by the SA113 and YUSA145 strains in the presence or absence of clemastine was identified by qPCR (targeting the chromosomal *gyrB* locus). The relative concentration of eDNA in biofilms of the SA113 and YUSA145 strains without clemastine exposure after 24 h (in terms of gyrB transcription level) was significantly higher than that of the control. The data presented was the average of three independent experiments (mean ± SD). Compared with control, *, *P* < 0.05; **, *P* < 0.01; ***, *P* < 0.001; (independent sample *t* test). MRSA, methicillin-resistant S. aureus. MSSA, methicillin-sensitive S. aureus.

The inhibition of the biofilm formation of six S. aureus strains (YuSA80, YUSA139, YuSA145, CHS350, CHS712, and CHS101) was investigated by a series of concentrations (0, 6.25, 12.5, 25, and 50 μM) of clemastine that could not affect bacteria planktonic growth using crystal violet staining, demonstrating the dramatic inhibition of biofilm formation of the 5 S. aureus strains by clemastine (50 μM) (Fig. 2). This finding was further confirmed in 10 MRSA isolates and 15 MSSA isolates measured by crystal violet staining. Moreover, the inhibition of clemastine (50 μM) on the biofilm formation of 1 MRSA isolate and 1 MSSA isolate were measured by confocal laser scanning microscope (CLSM) (Fig. 3A to C). Furthermore, clemastine (50 μM) could inhibit the biofilm formation at different stages of SA113 and SA145 but there was no effect on the CFU count, including the initial attachment at 3 h, irreversible attachment stage at 6 h, and maturation stage at 24 h (Fig. S3).

To investigate the effect of clemastine on the release of eDNA in the biofilm matrix of the S. aureus. The release of eDNA from SA113 and YUSA145 strains in the presence or absence of clemastine was identified by qRT-PCR (targeting the chromosomal *gyrB* locus). The relative amount of eDNA in biofilms of the SA113 and YUSA145 without clemastine treatment was 2.96 and 13.27-fold high compared to that of the clemastine-treated group after 24h of biofilm formation, respectively (*P* < 0.05), implying that clemastine inhibited the release of eDNA during S. aureus biofilm growth (Fig. 3D).

**FIG 4:**
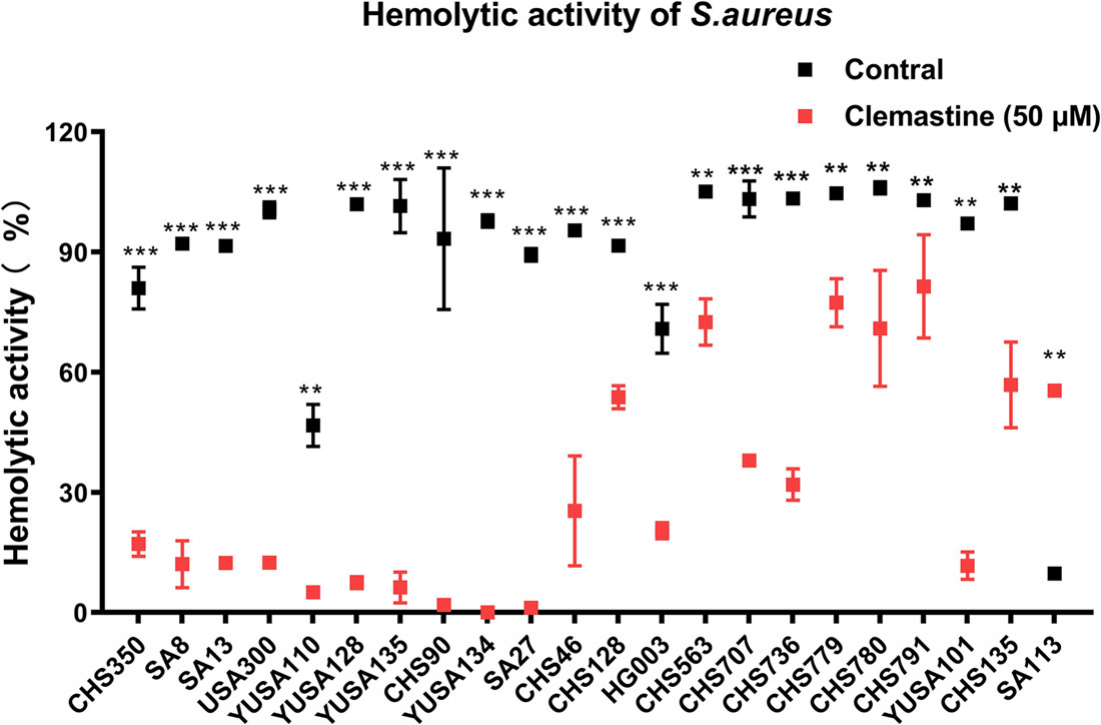
The reduced hemolytic activity of S. aureus by clemastine. Twenty-nine S. aureus strains were cultured with clemastine (50 μM) for 24 h then the supernatant was filtered through a 0.22 μm filter and incubated with 1% of rabbit erythrocytes at 37°C for 30 min. Subsequently, the OD_550_ of the mixture was measured. The hemolytic activity of 21 S. aureus clinical isolates was significantly reduced by clemastine. Compared with control, **, *P* < 0.01; ***, *P* < 0.001; n.s., not significant (independent sample *t* test).

The influence of clemastine on the hemolytic activity of S. aureus using rabbit erythrocytes was analyzed with the supernatant of 21 clinical S. aureus strains, indicating that the hemolytic activity was significantly reduced in most clinical S. aureus isolates (Fig. 4). Multiple reports have demonstrated the close relationship of the hemolytic activity with the α-hemolysin secretion of S. aureus. Furthermore, enzyme-linked immunosorbent assay (ELISA) was performed to determine the secretion levels of hla release from S. aureus clinical isolates (Fig. S4), suggesting clemastine exposure showed the unaffected change of α-hemolysin production in S. aureus.

To examine the mechanism of the clemastine in regulating S. aureus biofilm formation and hemolytic activity, we measured the transcription levels of several biofilm formation-related, virulence, and regulatory genes of SA113 via qRT-PCR in the absence and presence of clemastine (50 μM) at 24 h. The transcriptional levels of biofilm-related genes (*fnbB*, *icaA*, and *icaB*), virulence-related genes (*hlg*, *hld*, *lukde*, *lukpvl*, *beta-PSM*, *delta-PSM*, and *cap5A*), and the regulatory genes *agrA* were decreased at 24 h (Table 1). The reduction of hemolytic activity might be explained by the decreased expression of other hemolysins subtypes such as *hld*, *hlg*, *lukde*, *lukpvl*, *beta-PSM*, and *delta-PSM* (18).

**Table 1:** The transcriptional levels of transcriptional regulatory genes, biofilm formation related genes, virulence related genes after clemastine exposure for 24 h^*a*^

Gene name	SA113
	24 h
Transcriptional regulatory genes	
*agrA*	0.54 ± 0.157
*luxS*	1.14 ± 0.20
*sarA*	0.99 ± 0.26
*sigB*	1.48 ± 0.36
*saeR*	1.15 ± 0.32
Biofilm formation related genes	
*atl*	1.28 ± 0.40
*clfA*	2.46 ± 0.91
*fnbB*	0.02 ± 0.02
*icaA*	0.20 ± 0.05
*icaB*	0.39 ± 0.13
*cidA*	0.98 ± 0.33
*xdrA*	0.94 ± 0.32
Virulence related genes	
*hla*	3.71 ± 1.86
*hld*	0.66 ± 0.30
*hlb*	1.06 ± 0.24
*hlg*	0.40 ± 0.20
*lukde*	0.47 ± 0.05
*lukpvl*	0.07 ± 0.001
*beta-psm*	0.92 ± 0.30
*dedlta-psm*	0.61 ± 0.21
*cap5A*	0.57 ± 0.16

a Clemastine was used at 50μM. The RNA levels were detected by RT-qPCR, with untreated clone as the reference strain (RNA level = 1.0). The RNA levels of genes in clemastine treated clone was compared to the untreated clone.

### The genetic mutations in the clemastine-induced clone and the bioinformatics analysis.

**FIG 5:**
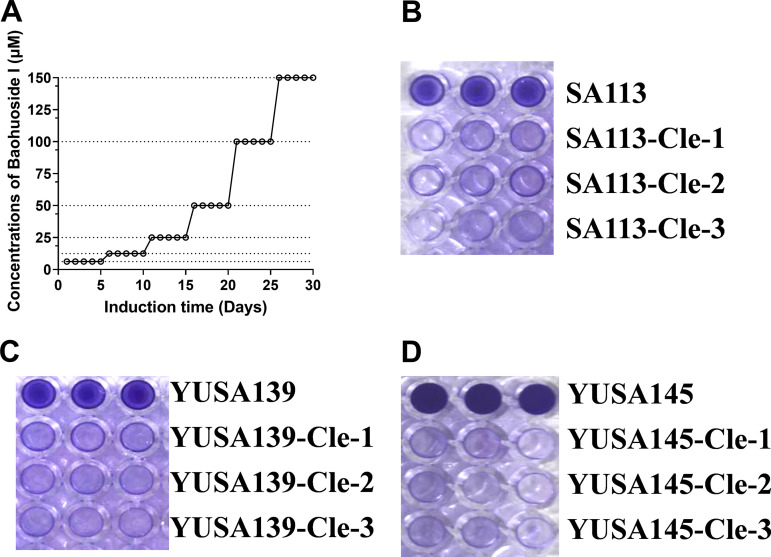
The *in vitro* induction of S. aureus by clemastine exposure and the biofilm formation of clemastine-induced tolerant derivatives. The liquid culture of YUSA139, YuSA145, and SA113 strains were consecutively induced under clemastine pressure from 50 μM until 300 μM. The induced concentration of clemastine was elevated with 50 μM every 5 days. After the 30-day (D30) induction, three individual clones of every parental strain of YUSA139, YuSA145, and SA113 were isolated, and their biofilm formation was determined compared with the untreated control by crystal violet staining. (A) The *in vitro* induction process of YUSA139, YuSA145, and SA113 strains. The biofilm formation was determined by crystal violet staining in (B) YUSA139 and its derivations with clemastine tolerance, (C) YuSA145 and its derivations with clemastine tolerance, (D) SA113 and its derivations with clemastine tolerance.

To evaluate the potential target site of clemastine, the clemastine-induced tolerant S. aureus clones were selected by *in vitro* serial passaging under the pressure of clemastine. After the consecutive induction by clemastine for 30 days, three clemastine-induced tolerant S. aureus clones, which survived in the 300 μM clemastine and derived from the parental derivative clones of YUSA139, YuSA145, and SA113 strains, were isolated and the diminished biofilm formation of these derivatives was further demonstrated in comparison to that of the parental isolates (Fig. 5). The genetic mutations in the clemastine-induced tolerant SA113 clones in comparison to the parental SA113 were determined by whole-genome sequencing.

Genetic mutations were found in eight coding genes of clemastine-induced tolerant SA113, including teichoic acids export ABC transporter ATP-binding subunit TagH, anthranilate synthase component I, biotin synthase BioB, cyclic di-AMP phosphodiesterase GdpP, amino acid adenylation domain-containing protein, acetyl-CoA carboxylase biotin carboxylase subunit, and a hypothetical protein (Table 2). Worthy of our attention, two genetic mutations located in GdpP of clemastine-induced tolerant SA113 were further validated through PCR and sanger sequencing in the chromosome YUSA139 and YuSA145, suggesting GdpP might be the potential target of clemastine.

**Table 2:** The genetic mutations between the SA113 parental isolates and its clemastine-induced tolerant T1 clone by the whole-genome sequencing^*a*^

Ref_gene_ID	Mutate type	NA mutations	AA mutations	Subject description
*SA113_GM000130*	Nonsyn	C742T	E248K	Teichoic acids export ABC transporter ATP-binding subunit TagH
*SA113_GM000378*	Nonsense	C370T	Q124X	Anthranilate synthase component I
*SA113_GM000779*	Nonsyn	G523A	A175T	Hypothetical protein
*SA113_GM001242*	Nonsyn	C573T	A191V	Biotin synthase BioB
*SA113_GM001742*	Nonsyn	T156C	L52S	Cyclic-di-AMP phosphodiesterase GdpP
*SA113_GM001742*	Syn	C1019T	I339I	Cyclic-di-AMP phosphodiesterase GdpP
*SA113_GM001857*	Nonsyn	C6513A	A2171E	Amino acid adenylation domain-containing protein
*SA113_GM002086*	Nonsyn	C192T	R64H	Acetyl-CoA carboxylase biotin carboxylase subunit

a The S. aureus SA113 clone was serially subcultured in TSB containingunder clemastine pressure from 50 μM until 300 μM with 50 μM increasing concentrations every 5 days. The individual clone was isolated from the 30-day (D30) induction SA113 strain and untreated control SA113 strain, and detected by the whole-genome sequencing. NA, nucleotide; AA, amino acid.

**FIG 6:**
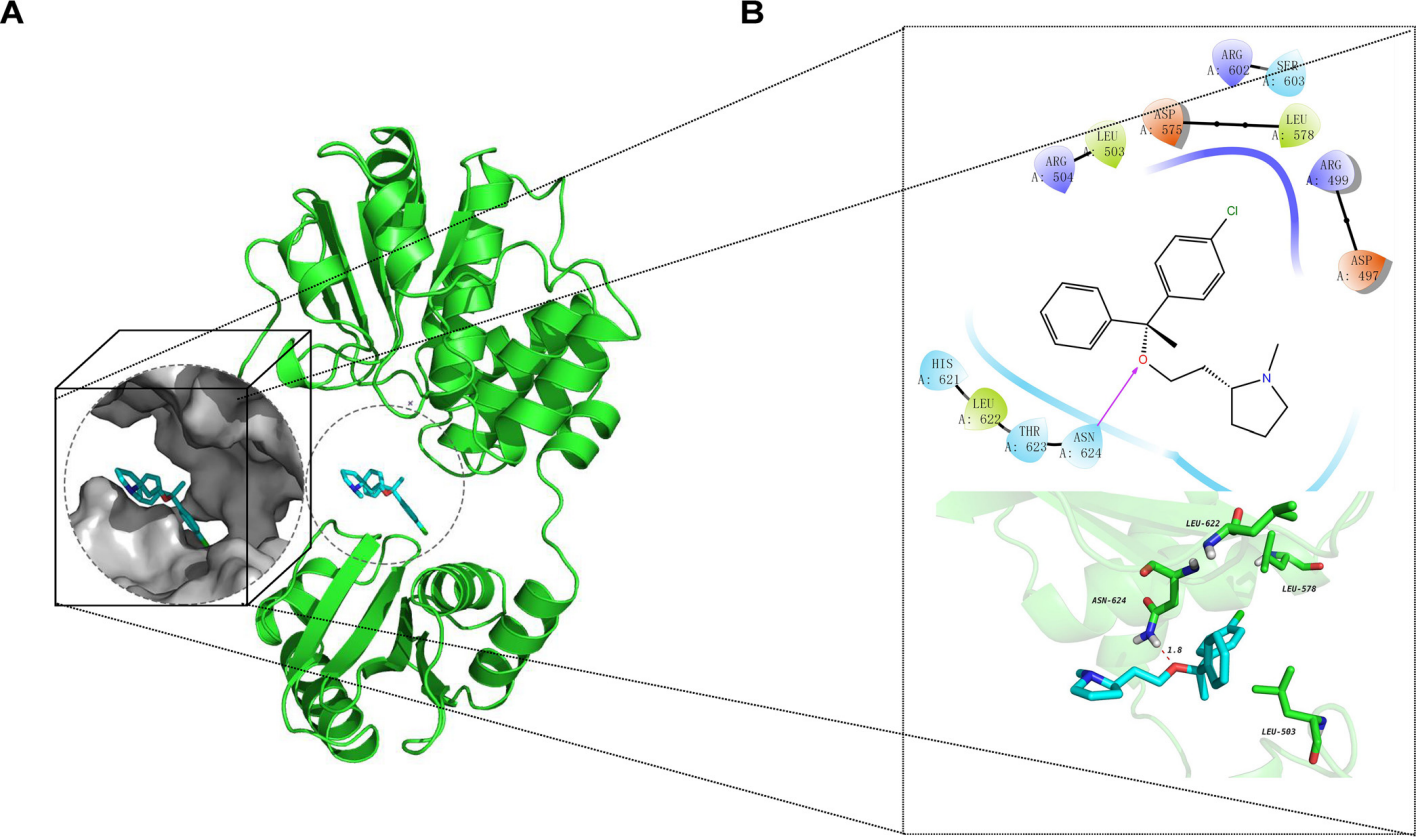
The molecular docking of GdpP protein and clemastine. High resolution three-dimensional docked structure of GdpP protein (green) and clemastine (blue) (A). The details of docked complexes show interactions between GdpP protein and clemastine (B). The clemastine is shown in blue stick presentation and colored for different elements (blue for nitrogen atom; red for oxygen; white for hydrogen; green for carbon skeleton of GdpP protein). The distances of hydrogen bonds are labeled with a dashed red line.

Multiple reports have demonstrated the important role of GdpP in the regulation of S. aureus growth and participation in biofilm formation and the antibiotic tolerance toward β-lactam/glycopeptide (19, 20). Therefore, the binding model of GdpP protein and clemastine was predicted by molecular docking analysis. Clemastine was molecularly docked with the cyclic di-AMP binding pocket of S. aureus GdpP protein (Fig. 6A), suggesting the oxygen atom could act as a hydrogen bond acceptor to form a hydrogen bond with ASN624, and the interatomic distances between hydrogen and oxygen were shown within 1.8 Å Furthermore, the compound of clemastine participates in a hydrophobic interaction with LEU622, LEU578, and LEU503 of S. aureus GdpP protein (Fig. 6B).

### Influence of *gdpP* gene overexpression on inhibition biofilm by clemastine.

**FIG 7:**
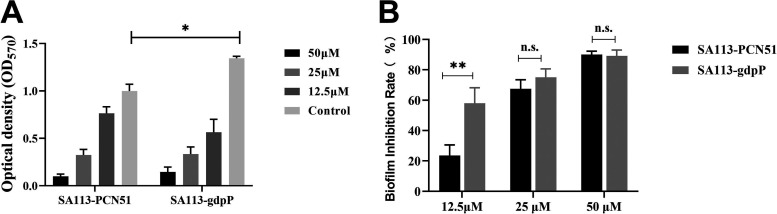
Inhibition of biofilm formation of S. aureus with *gdpP* overexpression by clemastine. With 2 μM CdCl_2_ induction, SA113 with *gdpP* overexpression and the control SA113 with empty pCN51 strains were incubated with clemastine 24 h at a series concentration of 0, 12.5, 25, and 50 μM. (A) The biofilm was then measured by crystal violet staining. The biofilm formation of SA113-pCN51 and SA113-gdpP overexpression was decreased by clemastine; however, (B) the biofilm inhibition rate of SA113-gdpP was higher than SA113-pCN51 at 12.5 μM clemastine. The data presented was the average of three independent experiments (mean ± SD). Compared with control, n.s., not significant; *, *P* < 0.05; **, *P* < 0.01; (independent sample *t* test).

To investigate the influence of GdpP on clemastine tolerance, the *gdpP* gene overexpression strain was constructed in the SA113 strain. The overexpression level of the *gdpP* gene was determined by qRT-PCR (Fig. S5). The inhibition quantification of biofilm formation in SA113-gdpP by 12.5 μM clemastine showed about 2-fold in comparison to that in the empty vector control (SA113-pCN51), suggesting the overexpression *gdpP* in SA113 markedly increased the anti-biofilm activity of clemastine (Fig. 7 and Fig. S5).

### Comparison of cyclic di-AMP levels and proteomics of S. aureus treated with clemastine.

To further understand and analyze the comprehensive impact of clemastine on S. aureus, the cyclic di-AMP level and proteomics effects of S. aureus after exposure to clemastine (50 μM) were evaluated using high-throughput mass spectrometry. The concentration of intracellular cyclic di-AMP was significantly increased in the clemastine-treated group compared with that in the untreated group at the equal weights cell pellets (Fig. S6).

**FIG 8:**
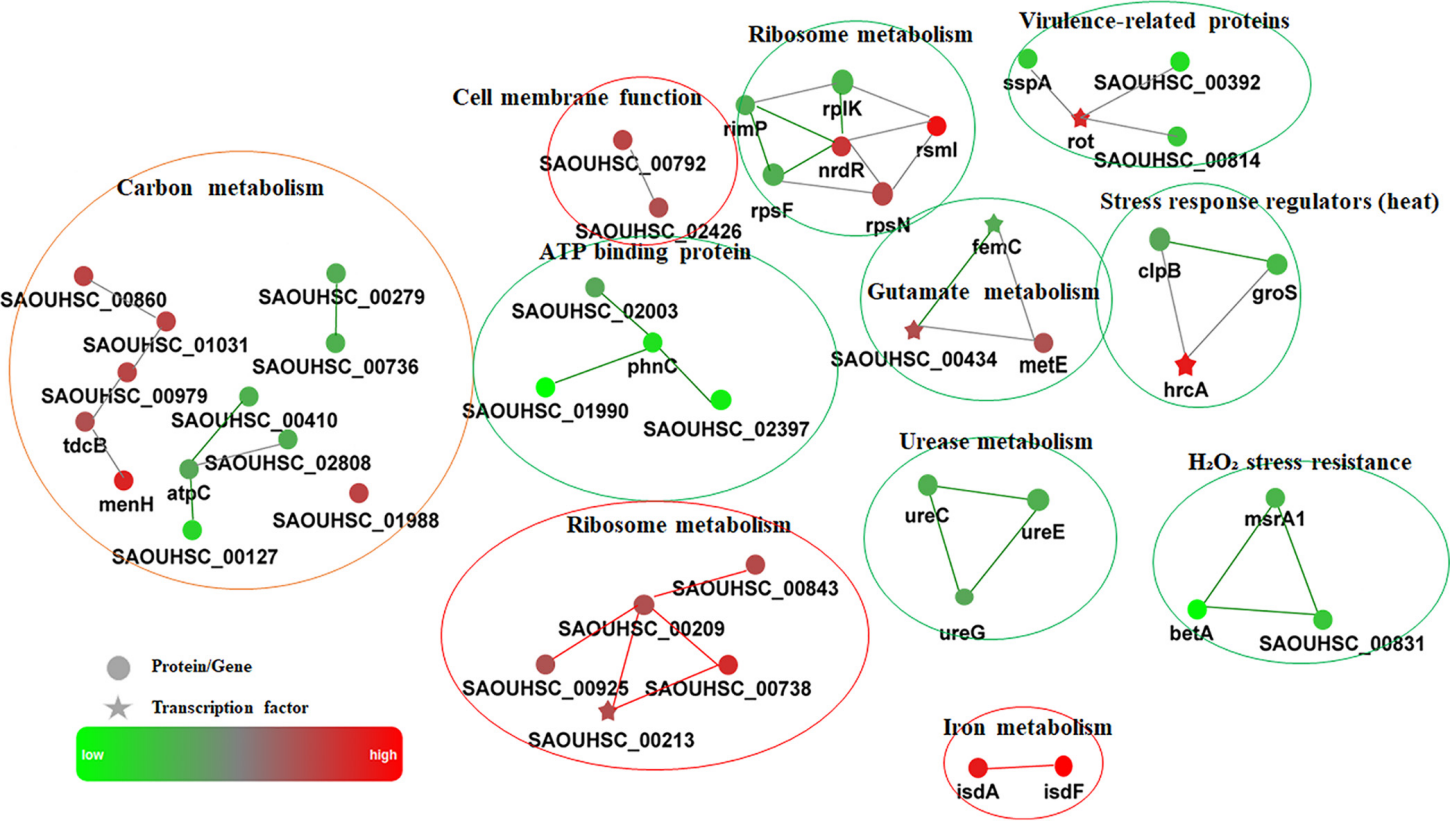
Protein-protein interaction network (PPI) of the proteomics data. Protein-protein interaction networks for the most representative proteins and signaling pathways influenced by half of the MIC clemastine treatment tested against S. aureus. Upregulated or downregulated proteins are indicated in red or green, respectively. The lines represent protein-protein interactions, including binding/association, phosphorylation, activation, and inhibition.

In total, 1600 proteins, including a significant decrease of 39 proteins and a significant elevation of 34 (*P* < 0.05), were identified in the clemastine-treated group compared with the control (DMSO-treated) group. The functional analysis of the biological process, molecular function, cellular component, and protein-protein interaction network (PPI) were constructed according to the gene ontology (GO) and KEGG pathway database (Fig. S7 and S8). The proteomic analysis revealed a significant enrichment with downregulated virulence-related proteins, stress response regulators (heat and oxide), ATP-binding proteins, and urease metabolism, and upregulated glutamate metabolism, iron metabolism, and ribosome metabolism (Fig. 8 and Table 3).

**Table 3:** Proteins expressed differently (up- or downregulation) in S. aureus with clemastine exposure

Protein name	Fold change	Description
Virulence-related proteins		
Rot	3.71	Global regulator with both pitive and negative effects that mediates modulation of several genes involved in virulence.
SAOUHSC_00392	0.19	Staphylococcal superantigen-like 7
SAOUHSC_00814	0.28	Truncated secreted von Willebrand factor-binding protein (Coagulase) VWbp, putative
SspA	0.26	Glutamyl endopeptidase
Gutamate metabolism		
FemC	0.47	Factor involved in methicillin resistance / Glutamine synthetase repressor
MetE	2.00	5-methyltetrahydropteroyltriglutamate--homocysteine methyltransferase
SAOUHSC_00434	2.11	Transcription activator of glutamate synthase operon
Stress response regulators (heat)		
HrcA	4.66	Heat-inducible transcription repressor HrcA
ClpB	0.50	Chaperone protein ClpB
GroS	0.36	Chaperonin Binds to Cpn60 in the presence of Mg-ATP and suppresses the ATPase activity of the latter
H2O2 stress resistance		
SAOUHSC_00831	0.30	Organic hydroperoxide resistance protein-like
MsrA1	0.38	Peptide methionine sulfoxide reductase MsrA 1
BetA	0.10	Oxygen-dependent choline dehydrogenase
Urease metabolism		
UreE	0.42	Urease accessory protein UreE
UreC	0.42	Urease subunit alpha
UreG	0.50	Urease accessory protein UreG
ATP binding protein		
SAOUHSC_02003	0.49	Putative multidrug export ATP-binding/permease protein SAOUHSC_02003
PhnC	0.17	Phphonates import ATP-binding protein PhnC
SAOUHSC_01990	0.10	Amino acid ABC transporter, ATP-binding protein, putative
SAOUHSC_02397	0.14	ABC transporter, ATP-binding protein, putative
Iron metabolism		
IsdA	4.69	Iron-regulated surface determinant protein A
IsdF	6.92	Iron-regulated surface determinant protein F
Ribosome metabolism		
NrdR	3.03	Negatively regulates transcription of bacterial ribonucleotide reductase nrd genes and operons by binding to NrdR-boxes
RplK	0.39	50S ribomal protein L11
RpsF	0.43	30S ribomal protein S6
RimP	0.44	Ribome maturation factor
RsmI	5.35	Ribomal RNA small subunit methyltransferase I
RpsN	2.33	30S ribomal protein S14
Sbstrate-specific transporter activity		
SAOUHSC_00209	2.07	PTS system, gluce-specific IIBC component, putative
SAOUHSC_00213	2.16	EIIA. The phosphoenolpyruvate-dependent sugar phosphotransferase system (sugar PTS) catalyzes the phosphorylation of incoming sugar substrates concomitantly with their translocation across the cell membrane.
SAOUHSC_00738	3.56	MFS domain-containing protein
SAOUHSC_00843	2.25	ABC transporter permease
SAOUHSC_00925	2.00	ABC transporter domain-containing protein
Carbon metabolism		
AtpC	0.49	ATP synthase epsilon chain
SAOUHSC_02808	0.44	Gluconate kinase
SAOUHSC_00127	0.21	Cap5N protein/UDP-gluce 4-epimerase, putative
SAOUHSC_00279	0.45	Cystatin-like fold lipoprotein
SAOUHSC_00410	0.42	GTP-binding protein
SAOUHSC_00736	0.41	Putative lipid kinase SAOUHSC_00736
SAOUHSC_00860	2.46	Trifunctional nucleotide phosphoesterase protein
SAOUHSC_01031	2.45	Cytochrome d ubiquinol oxidase, subunit I, putative
TdcB	2.07	Catalyzes the anaerobic formation of alpha-ketobutyrate and ammonia from threonine in a two-step reaction
SAOUHSC_00979	2.39	Acetyltransferase (GNAT family)
MenH	4.03	Putative 2-succinyl-6-hydroxy-2,4-cyclohexadiene-1-carboxylate synthase
SAOUHSC_01988	2.48	Putative tRNA (cytidine(34)-2'-O)-methyltransferase
Cell membrane function		
SAOUHSC_00792	2.41	Cell division inhibitor
SAOUHSC_02426	2.07	Membrane protein, putative
Uncharacterized protein		
SAOUHSC_02458	0.26	DUF3885 domain-containing protein
SAOUHSC_02436	2.68	Uncharacterized protein
SAOUHSC_02100	2.03	DUF2154 domain-containing protein
SAOUHSC_01966	2.22	Uncharacterized protein
SAOUHSC_00030	2.20	Uncharacterized protein
SAOUHSC_01475	4.20	Uncharacterized protein
SAOUHSC_02689	0.29	Uncharacterized protein
SAOUHSC_00052	0.48	Uncharacterized lipoprotein
SAOUHSC_00061	4.92	Uncharacterized protein
SAOUHSC_00146	2.31	Uncharacterized protein
SAOUHSC_00377	0.48	Uncharacterized protein
SAOUHSC_00618	2.55	Uncharacterized protein
SAOUHSC_00660	0.27	Uncharacterized protein
SAOUHSC_00890	3.78	Uncharacterized protein
SAOUHSC_00949	0.50	Uncharacterized protein
SAOUHSC_01073	2.19	Uncharacterized protein
SAOUHSC_01130	2.93	Uncharacterized protein
SAOUHSC_01761	3.86	Uncharacterized protein
SAOUHSC_01872	0.28	Uncharacterized protein
SAOUHSC_02376	2.13	Uncharacterized protein
SAOUHSC_02604	0.46	Uncharacterized protein
SAOUHSC_02755	0.38	Uncharacterized protein
SAOUHSC_03034	2.10	Uncharacterized protein
SAOUHSC_01572	2.03	Conserved hypothetical phage protein
SAOUHSC_02028	2.16	PhiETA ORF57-like protein
SAOUHSC_02049	4.69	Phage terminase, large subunit, PBSX family

### Thermal stability assay of GdpP protein treated with clemastine.

**FIG 9:**
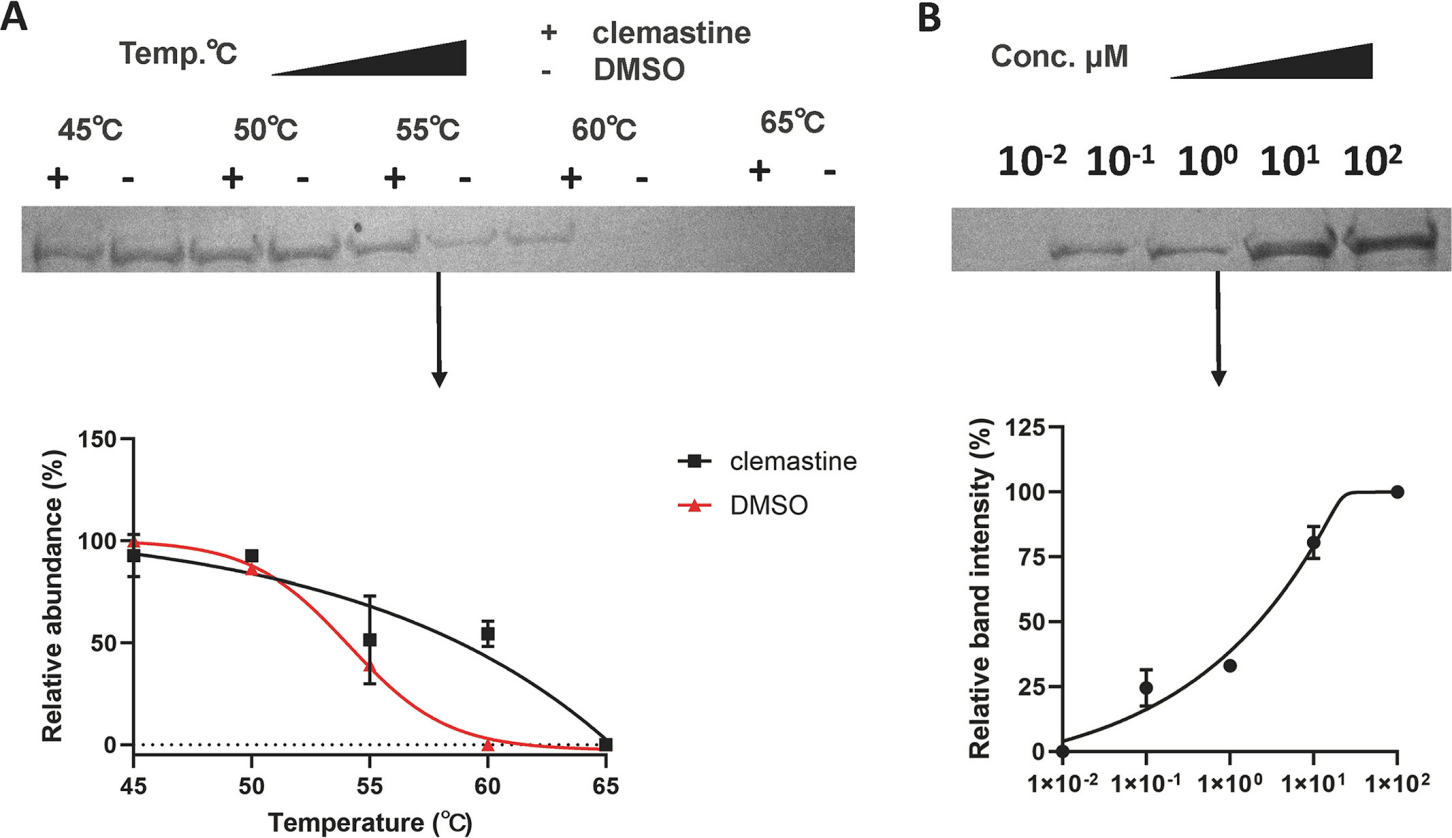
The thermal stability melt curve and dose-response curve. (A) The thermal stability melt curve. The GdpP protein and clemastine (10 μM) were incubated and heated individually at different temperatures. The supernatants were centrifuged and analyzed by SDS-PAGE. (B) Dose-response curve. The GdpP protein and various concentrations of clemastine were incubated and heated at 60°C and analyzed by SDS-PAGE. Protein-abundance graph derived from SDS-PAGE. Data are representative of two independent experiments (*n* = 2).

The ΔTm of thermal shifts assay has been verified to correlate with inhibition efficacy (IC_50_) by other methods and is widely used for the validation of the interaction between pathetical target protein and drugs (21–23). Therefore, the GdpP protein with His-tagged was successfully expressed and harvested. The interaction between the GdpP protein and clemastine was tested using the thermal stability assay under the condition of different heating temperatures or various concentrations of clemastine. Our data showed increased thermal shifts in the clemastine (10 μM) adds group under different temperatures, and the thermal stability of GdpP protein upon treatment at 60°C with increasing concentrations of clemastine, demonstrating that clemastine increased the thermally stabilized of GdpP protein by binging to GdpP protein (Fig. 9A and B).

## DISCUSSION

S. aureus has become one of the major causes involved in a wide range of community- or hospital-acquired infections. Chronic or persistent S. aureus infection has posed a serious threat to human health due to recalcitrant biofilms formation which is highly tolerant of traditional antibiotics (24, 25). Clemastine is one of the first-generation antihistamines with slight side effects, such as drowsiness. Recent studies found clemastine might be used as the remyelinating therapy of multiple sclerosis and seldom reports have demonstrated the inhibition of clemastine on the multiple growth stages of plasmodium parasite and mycobacterial growth (25–27). These studies strongly suggest the complicated physiological function of clemastine against a variety of human diseases, while data from studies addressing clemastine for the chemotherapy of S. aureus infection are still lacking.

In this study, the planktonic growth of S. aureus was unaffected by clemastine with a concentration of ≤200 μM, however, the inhibition of S. aureus growth could be enhanced by the subinhibitory concentrations of oxacillin when combined with 50 μM clemastine. *M*ethicillin or oxacillin-resistant S. aureus (MRSA) often means their resistance toward almost all available β-lactam antibiotics and have just limited available choices for the treatment of S. aureus infection, including vancomycin, linezolid, and daptomycin (2, 3). Clemastine can enhance the β-lactams susceptibility in S. aureus, suggesting the combination of β-lactams and clemastine might become a novel treatment strategy against S. aureus infections by the dosage reduction of β-lactam antibiotics. The glutamine synthetase is positively related to methicillin resistance due to involvement in the amidation of the iso-d-glutamate of the peptidoglycan stem peptide (28). The proteomics data showed that the FemC (glutamine synthetase repressor), which is a factor involved in methicillin resistance, were downregulated, and MetE (transcription activator of glutamate synthase operon) and SAOUHSC_00434 (5-methyl-tetrahydro pteroyl triglutamate–homocysteine methyltransferase) were upregulated after clemastine treatment, indicating that the action mechanism of clemastine was similar to methicillin. This may be associated with the synergistic effect of clemastine with oxacillin on the planktonic growth of S. aureus. In addition to the changes of glutamine synthetase, our data suggested influence bacteria growth due to impaired cell division and translational function that of S. aureus by clemastine, because of the transcription of SAOUHSC_00792 (cell division inhibitor), ribosomal protein genes NrdR (a negative regulator of ribonucleotide transcription), RsmI, and RpsN were increased, and RimP, RpsF, and RplK were decreased. Particularly interesting, the planktonic growth of S. agalactiae, E. faecalis, A. baumannii, and E. coli can also be inhibited by clemastine with a concentration of ≥100 μM similar to that in S. aureus. Therefore, the clinical significance of the sight inhibition of clemastine on the planktonic growth of S. aureus and other bacteria needs to be further studied.

Biofilm formation and hemolysin participate in the pathogenesis of S. aureus infections (5, 29). In this study, the robust inhibition of biofilm formation and hemolytic activity by 50 μM clemastine could be found in S. aureus. Moreover, the decreased transcriptional level of biofilm formation relevant genes (*fnbB*, *icaA*, and *icaB*) and virulence genes (*hlg*, *hld*, *lukde*, *lukpvl*, *beta-PSM*, *delta-PSM*, and *cap5A*), and the regulatory genes *agrA* were determined by qRT-PCR in SA113 at 24 h. The *agr* quorum-sensing system is a positive regulator of virulence (*hla*, *hlb*, PSM cytolysin genes, and *al*). The reduced hemolytic ability could be due to the inhibition of *agr* quorum-sensing system by clemastine, this is consistent with the result of proteomics data that S. aureus treatment with clemastine decreased the SspA, superantigen-like 7 protein, and VWbp binding protein. Due to the biofilm having a complex and dynamic intracellular environment (low-oxygen, low-pH and, low-nutrient conditions), the upregulation of stress-response regulons and repression of urease operons were observed in the S. aureus biofilm states (30, 31). Consistent with the phenotype of inhibition biofilm formation, proteomic analysis HrcA (heat-inducible transcription repressor) was upregulated, and ClpB, GroS (heat shock proteins), UreE, UreC, and UreG (urease accessory protein) were downregulated in the clemastine treatment group in contrast to the DMSO treatment group. Three proteins resistant to H_2_O_2_ stress were downregulated, which were BetA (oxygen-dependent choline dehydrogenase), SAOUHSC_00831 (organic hydroperoxide resistance protein-like), and MsrA (Peptide methionine sulfoxide reductase) (32). Overall, clemastine provides a dual role in preventing the pathogenesis of S. aureus by suppressing hemolytic activity and biofilm formation.

Through the *in vitro* induction of clemastine and whole-genome sequencing, the GdpP protein was found to be the potential target site of clemastine against S. aureus. Furthermore, the binding modes of clemastine and GdpP protein can be successfully predicted and established by molecular docking programs. More importantly, the overexpressing *gdpP* in SA113 significantly enhances the inhibition capacity of clemastine against biofilm formation, suggesting that *gdpP* might be the potential target of clemastine to inhibit the biofilm formation. GdpP protein encodes a phosphodiesterase to hydrolyze the cyclic di-AMP into 5′-phosphadenylyl-adenosine (pApA) or two molecules of AMP. The cyclic di-AMP is crucial as a second messenger for S. aureus growth and its intracellular overdose accumulation can result in multiple functional disorders (33–35). The loss of GdpP protein leads to cell death in Bacillus subtilis and Mycoplasma pneumoniae due to cyclic di-AMP intracellular accumulation (33, 36). S. aureus biofilm formation markedly defective accompanied by high levels of cyclic di-AMP was caused by a *gdpP* mutation (10). T The disruption of the *gdpP* gene abolished the secretion of hemolysin from S. aureus and Streptococcus suis (20, 29). Besides, loss of function of S. aureus GdpP protein could lead to β-lactam/glycopeptide tolerance (19, 37). Therefore, the concentration of cyclic di-AMP in the cells of S. aureus was further quantified via mass spectrometry and the thermal stability assay of GdpP protein treated with clemastine was performed in this study. The concentration of cyclic di-AMP increased in the clemastine-treated group compared with the untreated group. The clemastine increased the thermally stabilized GdpP protein by binging to GdpP protein. In total, the interaction analysis between GdpP protein and clemastine in this study, including the molecular docking, *gdpP* overexpression experiment, and thermal stability assay supported that clemastine exerts its effects through targeting GdpP protein in S. aureus. However, the binding site, action mode, and specific mechanism of clemastine-GdpP interaction still need further investigation.

Conclusively, the clemastine may display a synergistic effect with oxacillin on planktonic growth of S. aureus. Moreover, the biofilm formation and hemolytic activity of clinical S. aureus isolates could be significantly inhibited by clemastine. The qPCR and proteomics analysis of SA113 treated with clemastine displayed the decreased transcriptional level of the biofilm formation relevant genes (*fnbB*, *icaA*, and *icaB*), virulence genes (*hlg*, *hld*, *lukde*, *lukpvl*, *beta-PSM*, *delta-PSM*, and *cap5A*), and the regulatory genes *agrA*, and decreased virulence-related proteins, stress response regulators proteins (heat and oxide), ATP-binding proteins, and urease metabolism, and increased glutamate metabolism, iron metabolism, and ribosome metabolism. Using the experiments of drug-induced genetic mutation and overexpression mutation gene, the GdpP protein might be the target site of clemastine in S. aureus. Further studies showed that clemastine docked with the cyclic di-AMP binding pocket of Staphylococcus aureus GdpP protein via molecular docking model, the concentration of cyclic di-AMP was higher in the clemastine-treated group than the untreated group via mass spectrometry, and the thermally stabilized of GdpP protein increased by binging to clemastine though the thermal stability assay, indicated that clemastine might exert its effects through targeting GdpP protein. The interaction of GdpP with clemastine in S. aureus and the clinical application of clemastine in the treatment of S. aureus infection warrants further investigations.

## MATERIALS AND METHODS

### Bacterial strains, growth conditions, antibiotics, and chemicals.

S. aureus SA113, Mu50, and NCTC 8325 strains were purchased from American type culture collection (ATCC). The 25 clinical isolates of S. aureus, including 15 MSSA isolates and 10 MRSA isolates, 2 E. faecalis isolates, and one isolate of S. agalactiae, A. baumannii, K. pneumoniae isolate, and E. coli, respectively, were collected from Shenzhen Nanshan People's Hospital and used in this study. The species identification of these bacteria isolates was performed by MALDI-TOF mass spectrometry (IVD MALDI Biotyper, Bruker, Karlsruhe, Germany). S. aureus, E. faecalis, and S. agalactiae strains were grown in tryptic soy broth (TSB) (OXOID, Basingstoke, UK) at 37°C. A. baumannii, K. pneumoniae, and E. coli strains were grown in Luria broth (LB) (1% tryptone, 0.5% NaCl, and 0.5% yeast extract) at 37°C. Oxacillin (catalog no. HY-B0465), vancomycin (catalog no. HY-B0671), linezolid (catalog no. HY-10394), and clemastine (catalog no. HY-B0298A) were purchased from MedChem-Express (MCE, Shanghai, People’s Republic of China). The dimethylsulfoxide (DMSO) was used to dissolve clemastine and equal volumes of DMSO were used as vehicle control in this study. The highest DMSO concentration in the incubation medium was 0.5%, which did not affect bacterial growth and biofilm formation (38).

### Growth curve of the bacteria strains.

The different bacteria strains were diluted 1:200 in TSB and grown 12 h to stationary-phase at 37 °C, 220 rpm. Bacterial growth curves were detected by Bioscreen C (Turku, Finland). The bacteria were grown in TSB with diverse concentrations (25, 50, 100, and 200 μM) of clemastine or other antimicrobial agents at 37°C shaking at 220 rpm. Bacterial growth curves in TSB without clemastine were used as an untreated control. Optical density at 600 nm (OD_600_) was measured at 1 h intervals for 24 h and drawn the growth curve. All experiments were repeated in triplicate at least three times.

### The inhibitory activity of bacteria biofilm formation by clemastine.

The inhibition of biofilm formation by clemastine was performed according to previously reported (39, 40). The S. aureus isolates were inoculated individually into 96 polystyrene microtiter plates with TSBG (TSB with 0.5% glucose) containing various concentrations of clemastine (0, 6.25, 12.5, 25, and 50 μM). At different stages of biofilm formation (initial attachment, 3 h; irreversible attachment stage, 6 h; and maturation stage, 24 h), the biofilm formation was determined using crystal violet staining measured at 570 nm with a spectrophotometer and the growth of planktonic cells was determined using CFU count. All experiments were repeated in triplicate at least three times.

### CLSM of biofilms.

Each S. aureus strain (1:200 dilution) was inoculated in 2 mL TBSG for 24h 37°C, with a cell culture dish inlaying a glass coverslip (World Precision Instruments, USA). To harvest the biofilms, the medium was aspirated off and the biofilm was washed three times with saline. For microscopy, the bacteria in a biofilm were stained with LIVE/DEAD reagents (1 μM SYTO9 and 1 μM propidium iodide [PI]; Thermo Fisher Scientific, Houston, TX) for 20 min in the dark. Confocal images were acquired using a Confocal Laser Scanning Microscope (FV3000, OLYMPUS, Japan) with a 60× oil immersion objective. Data images were acquired using Leica LAS AF software. Three-dimensional images were generated using MARIS (version 7.0.0) software (Bitplane).

### Isolation and quantification of (eDNA).

The isolation and quantification of extracellular DNA (eDNA) from the biofilms was performed as described previously with minor modifications (40, 41). Briefly, 24 h old biofilms cultured treated with clemastine or DMSO (200 μL each well) in a 96-well polystyrene plate each well was added 1 μL EDTA (0.5 M), chilled at 4°C for 1 h and recorded the initial OD_600_ value by a microplate reader. After measurement, 150 μL eDNA extraction solution (50mM Tris-HCl, 10mM ETDA, 500mM NaCl [pH 8.0]) was added to the wells. The biofilms were scraped off and centrifuged (18,000 × *g*) in precooling EP tubes for 10 min at 4°C. The eDNA in the supernatant was extracted with phenol-chloroform-isoamyl alcohol (25:24:1), precipitated with 100% alcohol and sodium acetate (NaOAc, 3 M, pH 5.2), and resuspended in Tris-EDTA buffer. The quantification of eDNA was performed by a NanoDrop 2000 spectrophotometer (Thermo Fisher Scientific, Waltham, MA) and qPCR with SYBR Premix Ex Taq (TaKaRa Bio, Inc., Shiga, Japan) using the primers specific for gyrB (gyrase B gene). For data processing, the y-axis, which represents the eDNA amount, equals to absolute quantitative value of eDNA/OD_600_. And the x-axis represents the Ct value of the *gyrB* gene.

### Hemolytic activity assay and the enzyme-linked immunosorbent assay (ELISA).

The assessment of hemolytic activity impacted by clemastine was performed as previously described (42). S. aureus strains were incubated in TSB with diverse concentrations of clemastine at 37°C for 24 h and the supernatant of bacterial culture was harvested by centrifugation. Subsequently, the bacteria in the supernatant were further removed with a 0.22 μm filter (Millipore). Commercialization 4% rabbit erythrocytes (SBJ-RBC-RAB003, Sbjbio, China) stored in Alsevers solution were diluted to 1% using phosphate-buffered saline (PBS). The sterile supernatant was mixed with 1% of rabbit erythrocytes according to a volume ratio of 1:1. The mixture was then incubated at 37°C for 30 min, and the OD_550_ was measured by a spectrophotometer. The 0.1% Triton X-100 was used for the positive-control of 100% hemolysis and PBS served as the negative-control of 0%. All experiments were repeated in triplicate and the data from each well were counted according to previously reported (43).

The effects of clemastine on the supernatant expression level of α-hemolysin protein released from S. aureus strains were further investigated. S. aureus strains were inoculated individually into 96-well polystyrene microtiter plates with TSBG containing 50 μM of clemastine or DMSO. After 24 h of static incubation at 37°C, the supernatants were collected for the determination of α-hemolysin secretion level by ELISA kit (Shanghai Jianglai Industrial Limited by Share Ltd.) following the manufacturer’s protocol.

### RNA isolation and RT-qPCR.

To investigate the impact of clemastine on the transcriptional level of the biofilm-associated genes and virulence-related genes, the S. aureus SA113 strain was used for RT-qPCR analysis. The S. aureus strains during the exponential growth phase treated with clemastine or DMSO and incubated in TSB at 37°C for 24 h. The bacteria were harvested by centrifuging and then washed twice with cold saline. The bacterial cells were homogenized for 5 rounds using a Mini-Bead beater (Biospec, Bartlesville, OK, USA) at 4,800 rpm and then the RNA of the supernatant of the homogenized bacteria for qRT-PCR was extracted using RNeasy Minikit (QIAGEN, Hilden, Germany) according to the manufacturer’s instructions. The RNA samples were reverse transcribed to cDNA with the PrimeScript RT Reagent kit (TaKaRa Biotechnology, Dalian, People’s Republic of China). The qRT-PCR was conducted using the SYBR Premix Ex Taq II kit (TaKaRa Biotechnology, Dalian, People’s Republic of China) on the Mastercycler ep realplex system (Eppendorf). The primers for the biofilm-associated genes and virulence-related genes were shown in Table S1. All experiments were repeated in triplicate.

### *In vitro* induction and selection of clemastine tolerant S. aureus isolates.

S. aureus isolates (YUSA139, YuSA145, and SA113) were induced under the in vitro pressure of clemastine with the initial concentration from 50 μM with increasing 50 μM induction concentration every 5 days for 30 consecutive days until to 300 μM (38, 43, 44). Three individual derivative clones were picked and isolated on the 30th day for subsequent three consecutive generations without clemastine exposure in TSB plates. The biofilm formation of the derivative clones that induced and untreated with clemastine was detected as described above. During the induction, S. aureus clone which could survive in the 300 μM clemastine and lost or diminished the capacity for biofilm formation was defined as clemastine tolerant derivatives.

### Whole-genome sequencing of clemastine tolerant clones.

Clemastine tolerant SA113 clone was isolated as described above and showed diminished biofilm formation. The chromosomal DNA in the clemastine tolerant SA113 clones was extracted using the MiniBEST Bacteria Genomic DNA Extraction kit Ver.3.0 (Takara Biotechnology, Dalian, China) for whole-genome sequencing. A total amount of 1μg DNA per sample was used for whole-genome sequencing by Novogene Company (Beijing, China). Illumina PE150 sequencing data were mapped against the S. aureus NCTC 8325 (NCBI Reference Sequence accession no. NC_007795.1) strain reference genome in BWA MEM software (v0.7.5a) 2 with standard parameters. The whole-genome sequencing files of the clemastine tolerant SA113 clone were deposited in the NCBI database with the biosample accession SAMN18385247 and SAMN18385248 (BioProject accession number PRJNA715935). Using the MUMmer comparison software, the sample sequence was serially compared with the reference sequence the parenteral isolate SA113 with the biosample accession SAMN15745744 and SAMN15745745 and screened for differences. The BLAST, TRF, Repeatmask software was used to filter the SNPs (single nucleotide polymorphisms) placed in the repeat region. Finally, reliable SNP was obtained according to our previous reports (44, 45).

### The prediction of the binding model of clemastine by autodock vina.

Molecular structure data files for S. aureus GdpP protein (5XT3) were downloaded from the Protein Data Bank (PBD). Using the Protein Preparation Wizard module of Schrödinger software to hydrogenate, repair missing residues, optimize the structure, remove the B chain, and water molecules, and then perform energy optimization (OPLS2005 force field, RMSD is 0.3 Å). The small molecule ligand cyclic di-AMP was used as the center, and the receptor grid generation module was used to make the lattice file and the box size was set to 20 A × 20 A × 20 A. OPLS3 force field and RMSD of 0.30 Å were selected for energy minimization. LigPrep converts the two-dimensional format of clemastine (zinc no. ZINC402830) structures to three-dimensional structures. The Glide Extra Precision (XP) mode is used for the docking Clemastine and GdpP protein.

### Construction of the *gdpP* gene overexpression strain.

The *gdpP* gene overexpression strain was achieved by the E. coli-Staphylococcus shuttle vector pCN51. The *gdpP* gene was inserted downstream of the CdCl_2_-inducible promoter of pCN51 and then the pCN51 empty plasmid and pCN51: *gdpP* plasmid was transferred into the S. aureus by electroporation. The primers for the construction of the *gdpP* gene overexpression strain were shown in Table S1.

### Proteomic analysis of S. aureus treated with clemastine.

The S. aureus strain was treated with clemastine a biofilm inhibitory concentration (50 μM) of clemastine or equal volume DMSO 1 h after growing in TSB to OD_600_ ∼0.8 at 37°C. The bacteria were harvested by centrifugation at 3,000g for 5 min at 4°C and transferred to a precooled 2 mL screw-cap tube. The 1.5 volumes of acid-washed glass beads (1 mm) and RIPA lysis buffer were added. The bacteria were lysed by bead-beating at 4 °C using a cell disruption device at 6 m/s for 4 min and the total protein was quantified to 1 mg/mL with lysis buffer and 100 μg of S. aureus total protein was reduced with 10 mM DTT (Sigma-Aldrich Co., St. Louis, MO) for 1 h at 70°C, followed by alkylation using 50 mM iodoacetamide (IAA, Sigma-Aldrich) for 15 min at room temperature in the dark. The samples were then desalted and buffer-changed three times with 100 μL 0.5 M ammonium bicarbonate by using Amicon Ultra Centrifugal Filters (10 kDa cutoff; Millipore, Billerica, MA). The proteins were digested with trypsin (Promega, Madison, WI) at a ratio of 1:40 at 37°C overnight. The proteins were reconstituted in 30 μL of 0.1% formic acid, among which 4 μL of each sample was injected into an LC system (UltiMate 3000 RSLC) with a C18 precolumn (100 μm × 20 mm, Acclaim PepMap 100 C18, 3 μm), followed by separation using a C18 tip column (75 μm × 250 mm, Acclaim PepMap RSLC, 2 μm). The mobile phases A and B were composed of 0.1% formic acid and 80% acetonitrile in 0.1% formic acid, respectively. The elution system started with 5% B for the first 5 min, followed by a linear gradient from 5% B to 38% B in the next 85 min and from 38% B to 95% B in the next 2 min, maintained at 95% B for another 3 min at a flow rate of 300 nL/min. The column was coupled to Q Exactive Plus mass spectrometer equipped with the nanospray ionization (NSI) interface. MS1 scans were acquired over a mass range of300 to 1500 m/z with a resolution of 70,000 and the corresponding MS2 spectra were acquired at a resolution of 17500, collected for maximally 50 ms. All multiply charged ions were used to trigger massspectrometry-massspectrometry (MS-MS) scans followed by a dynamic exclusion for 30 s. Singly charged precursor ions and ions of undefinable charged states were excluded from fragmentation. The protein identification and quantification were performed using Proteome Discoverer 2.4 base with the Sequest HT against the Uniprot reference proteome of S. aureus (strain NCTC 8325/PS 47) reference proteome database (UP000008816.fasta; 2889 entries; downloaded on February 20, 2021). To reduce false-positive identification results, a minimum unused score of 1.3 (equivalent to 95% confidence) and a false discovery rate (FDR) less than 1% were required for all reported proteins. Based on a 95% confidence level, at least one unique peptide per protein group was required for identifying proteins, and two quantified peptides were required for quantifying proteins. A 2-fold cutoff value was applied to determine upregulated and downregulated proteins in addition to a *P* value of less than 0.05 in at least two technical replicates. Bioinformatics Analysis: The differentially expressed proteins were uploaded into the OMICSBEAN database (http://www.omicsbean.com [platform can be used through the temporary IP address: http://211.149.220.136:8000]) for gene ontology (GO) annotation, including biological process, cellular component, molecular function, and KEGG pathway analysis. The PPI networks were constructed by using Cytoscape software according to the KEGG database.

### High-performance liquid chromatography (HPLC).

Overnight cultures of S. aureus 1:200 were grown in TSBG at 37°C with or without 50 μM clemastine, and samples were prepared for analysis as described for c-di-GMP with slight modifications (10). Briefly, the equal weights cell pellets were washed twice with cold molecular grade water (Corning) and lysed by homogenized for 5 rounds using Mini-Bead beater (Biospec, Bartlesville, OK, USA) at 4,800 rpm. The samples were centrifuged at 21,000 × *g* for 5 min. Supernatants were removed and stored at −80 °C until HPLC analysis. Samples were run similar to c-di-GMP (46) on an AB SCIEX TRIPLE QUAD 4500MD and peaks were quantified at 260 nm.

### Expression and purification of GdpP recombinant proteins in E. coli.

The DNA fragment of GdpP was cloned into the BamH I and Xho I sites of a pET28a for His-tagged vector with the primers CGCGGATCCATGAATCGGCAGTCCACTAAG and CCGCTCGAGTCATGCATCTTCACTCCTAC, the construct was described previously (47). The E. coli strain BL21(DE3) carrying the pET28a-GdpP plasmid was incubated in LB medium at 37°C with shaking (at 220 rpm) to logarithmic growth phase, and then induced expression at 25°C for 14 h with 1 mM IPTG. Subsequently, cells were lysed by sonication and the GdpP recombinant protein was stored at −20°C. The GdpP recombinant protein was purified according to the product instruction using the Mag-Beads His-Tag Protein Purification (C650033, Sangon Biotech).

### Thermal stability assay.

The stabilization of protein-compound (GdpP-clemastine) interaction was evaluated as described previously (21–23). Various concentrations of clemastine and GdpP protein were incubated for 30 min at room temperature and heated individually at different temperatures for 3 min and then centrifuged at 15,000 × *g* for 30 min at 4°C. The supernatants were analyzed by sodium dodecyl sulfate-polyacrylamide gel electrophoresis (SDS-PAGE).

### Statistical analysis.

All the data were analyzed SPSS (Version 16.0, Chicago, IL, USA) using the independent sample t-test. *P* values <0.05 were considered statistically significant.

### Ethics statement.

All procedures involving human participants were performed in accordance with the ethical standards of Shenzhen University School of Medicine and with the 1964 Helsinki declaration and its later amendments, and this study was approved by the ethics committee of the Shenzhen University School of Medicine. For this type of study, formal consent is not required.

### Data availability.

The whole-genome sequencing files of the clemastine-tolerant SA113 clone were deposited in the NCBI database with the biosample accession SAMN18385247 and the reference sequence the parenteral isolate SA113 with the biosample accession SAMN15745744 and SAMN18385255. We declare that the data supporting the findings of this study are available within the paper and Supplemental Material. The raw whole-genome sequencing data were posted in the Sequence Read Archive (SRA) database under the BioProject accession number PRJNA715935.
