# Supplementary material for: Clemastine Inhibits the Biofilm and Hemolytic of Staphylococcus aureus through the GdpP Protein
Source: Microbiol Spectr. 2022 Mar 2;10(2):e00541-21. doi: 10.1128/spectrum.00541-21 (PMC8941875; doi:10.1128/spectrum.00541-21)
Supplement: SUPPLEMENTAL FILE 1 — Supplemental material. Download SPECTRUM00541-21_Supp_1_seq10.pdf, PDF file, 0.7 MB [file spectrum00541-21_supp_1_seq10.pdf]

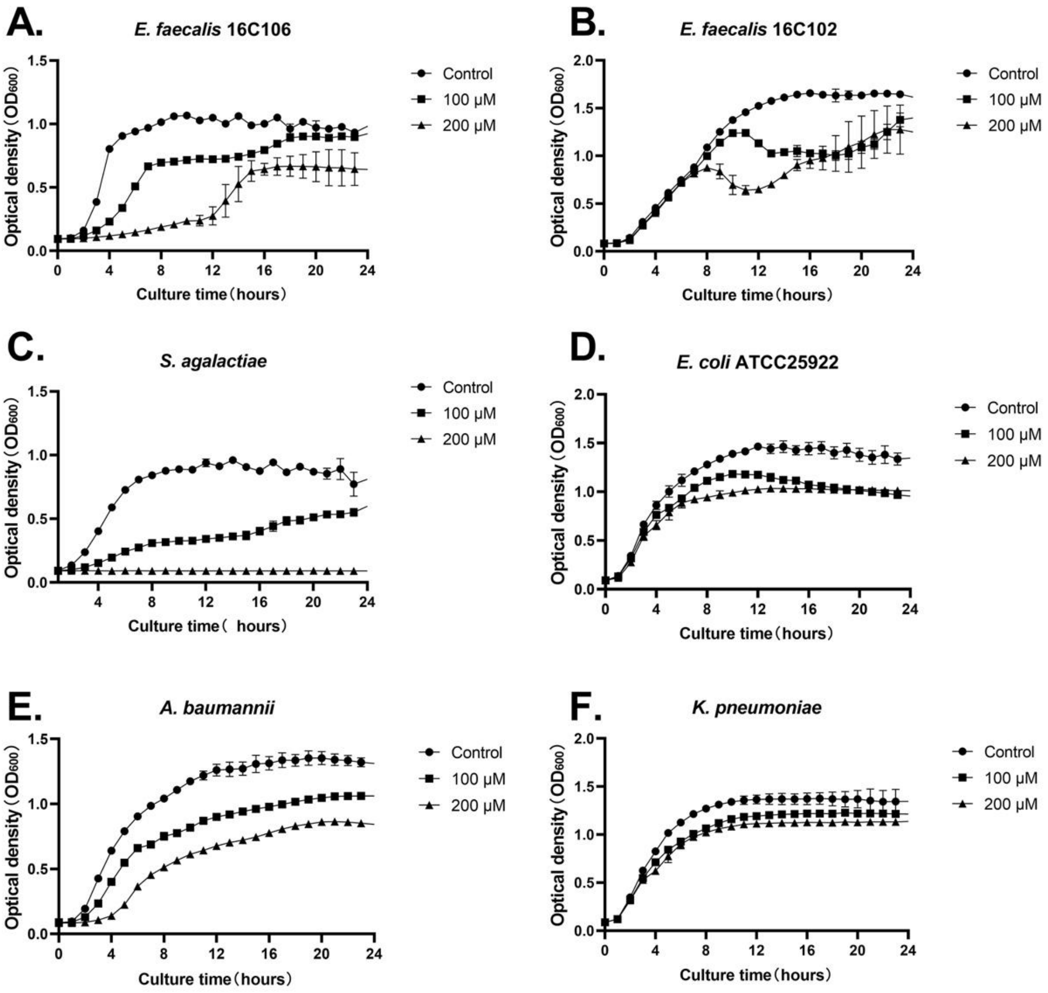

Figure S1. Clemastine inhibited the planktonic growth of *E. faecalis*, *S. agalactiae*, *A. baumannii*, *K. pneumoniae*, and *E. coli*. Overnight cultures (12 h) of the *E. faecalis*, *S. agalactiae*, *A. baumannii*, *K. pneumoniae*, and *E. coli* strains were diluted 1:200 into TSB containing clemastine (0, 100, 200 μM), then grown at 37°C with shaking at 220 rpm and monitored by measuring the OD_{600} at indicated time points, until 24 h. The experiments were repeated three times, and error bars indicate the standard deviation.

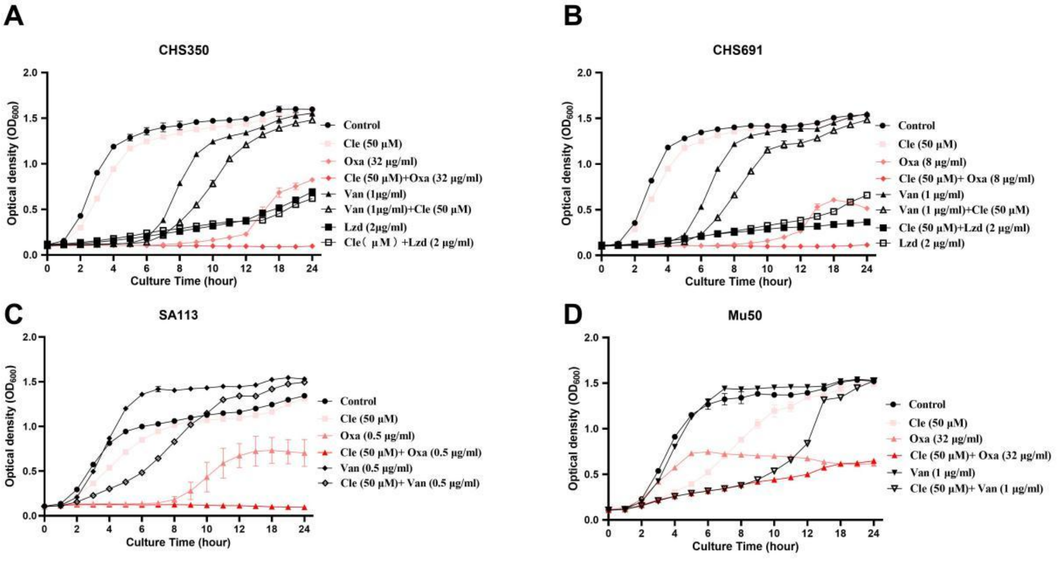

Figure S2. The synergistic effect of clemastine combined with linezolid, vancomycin, or oxacillin on the planktonic growth of *S. aureus*. Liquid cultures of the CHS350 (**A**)and CHS691(**B**), SA113(**C**), and CHS101(**D**) strains after 12 h were diluted 1:200 into TSB containing clemastine (50 μM) and co-incubated with single or combined with oxacillin, vancomycin, or linezolid, then grown at 37°C with shaking at 220 rpm and the growth curves were monitored by Bioscreen C measuring the OD_{600} at indicated time points, until 24 h. The experiments were repeated three times, and error bars indicate the standard deviation. The clemastine could enhance the inhibitory activity of subinhibitory concentration oxacillin (32 μg/ml, 8 μg/ml or 0.5 μg/ml) in MRSA CHS350, Mu50, CHS691 and MSSA SA113, while no synergistic effect with linezolid and vancomycin was found. Cle, clemastine; Oxa, oxacillin; Van, vancomycin, Lzd, linezolid.

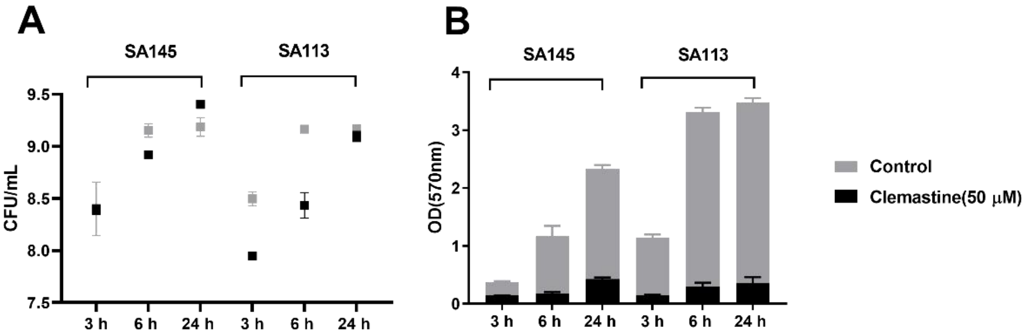

Figure S3. The effect of clemastine on the growth of planktonic *S. aureus* during biofim growth. In the presence or absence of clemastine, the SA113 and YUSA145 strains were statically cultured in TSB with 0.5% glucose. The bacterial counts were determined and biofilm formation was measured by crystal violet staining at three representative time points, including 3 h (initial attachment),6 h (irreversible attachment stage), and 24 h (maturation stage). The bacterial colonies were counted

and shown as CFU per 1 ml. **(A)** The CFU count and **(B)** Biofilm formation, suggesting clemastine reduced the biofilm formation in all three points and few notable differences were observed in the growth of planktonic bacteria. The experiments were repeated three times, and error bars indicate the standard deviation.

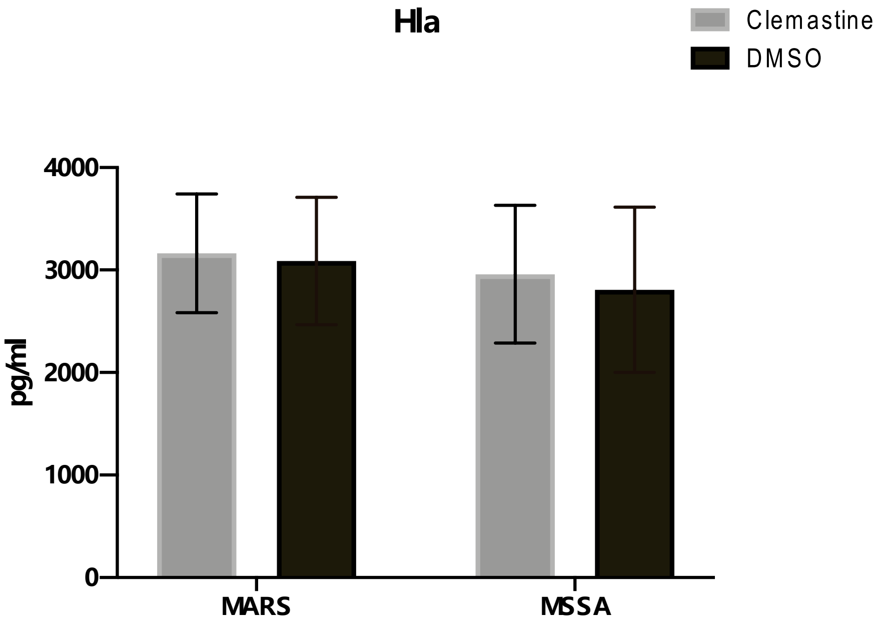

Figure S4. The effect of clemastine on α-hemolysin secretion in MARS and MSSA. The α-hemolysin secretion levels were quantified by ELISA at 24 h after Clemastine or DMSO incubate. (N=11 in MSSA; N=10 in MARS)

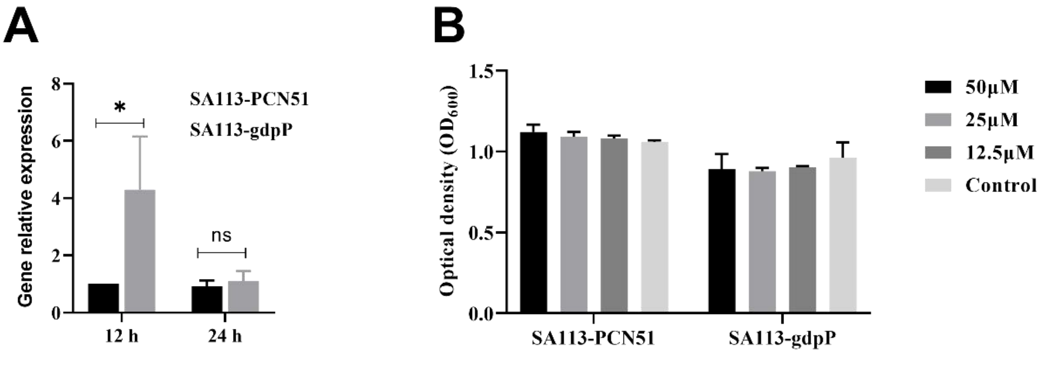

Figure S5. Influence of *gdpP* gene overexpression on growth by clemastine. (A) Overexpression of *gdpP* was evaluated by RT-qPCR. SA113-pCN51 and SA113-gdpP strains were incubated with 2 μM $CdCl_2$12h and24 h. Total cell lysates were used for the detection of *gdpP* over-expression and the transcriptional expression level of *gdpP* were significantly high compared with that in SA113-gdpP at 12 h. **(B)**With 2 μM $CdCl_2$ induction,SA113-pCN51 and SA113-gdpPstrains were incubated with clemastine 24 h at a series concentration of 0, 12.5, 25, 50μM. Then OD_{600}wasmeasured and no obvious change was found between SA113-pCN51 and

SA113-gdpP.The data presented was the average of three independent experiments (mean ±SD).

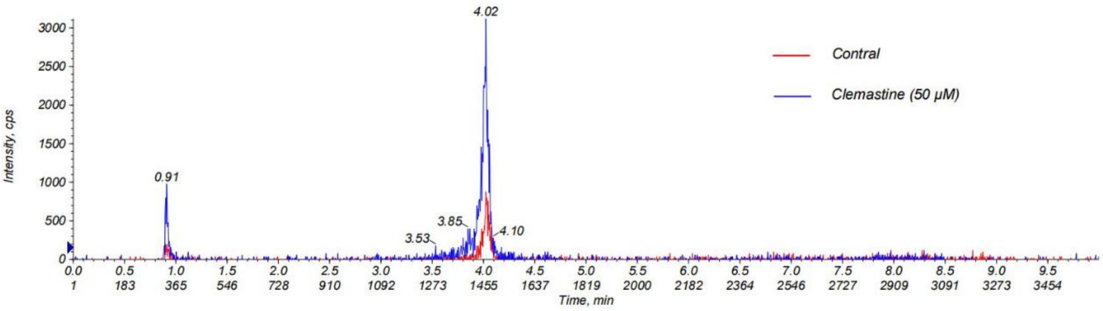

Figure S6. HPLC results for quantification of intracellular cyclic-di-AMP levels with clemastine exposure in *S .aureus* .The SA113 strain grown in TSBG with (Blue-line) and without (Red-line) clemastine (50μM) and the equal weights cell pellets were lysed and analyzed for intracellular cyclic-di-AMP levels via mass spectrometry after 24 h of bacteria growth.The abscissa represents the retention time (min) of cyclic-di-AMP detection, and the ordinate represents the intensity of the ion current (cps: count per second).

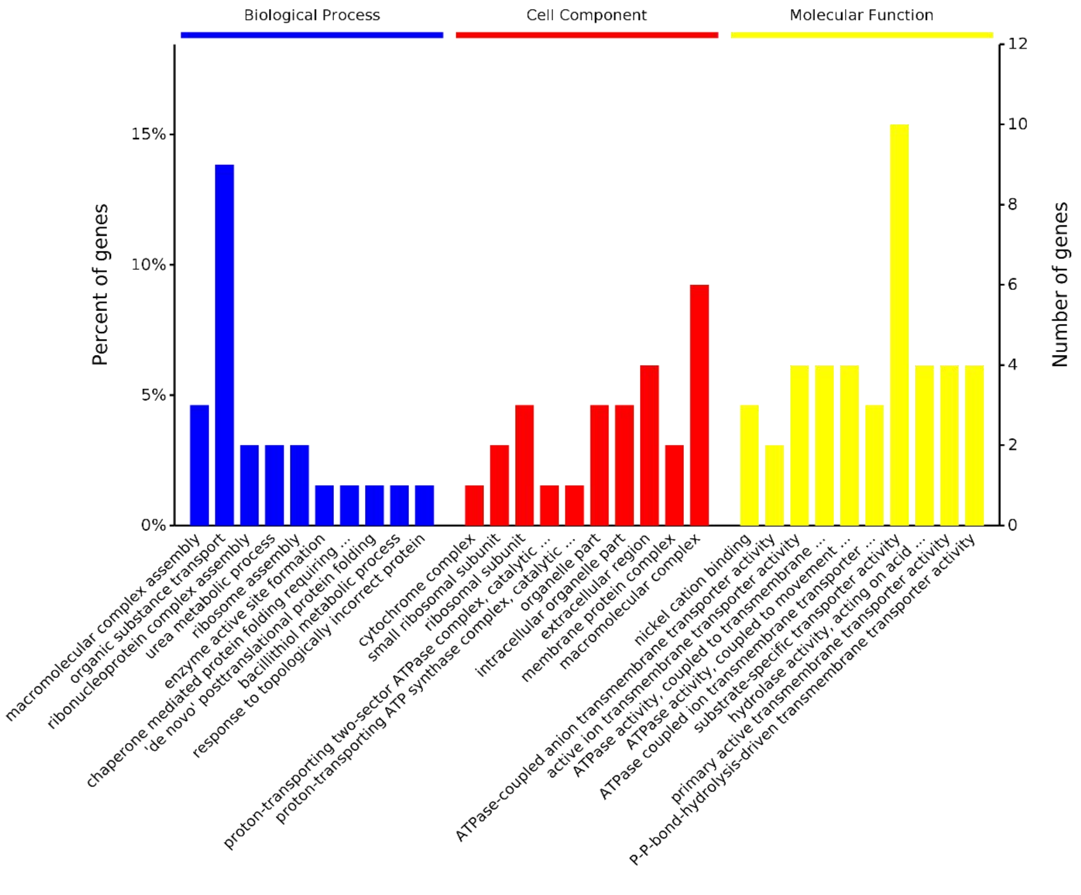

Figure S7. Biological process, cell component, and molecular functions of identified differentially expressed proteins (up- and downregulations) from 50 μMclemastine treatment tested against *S. aureus*.

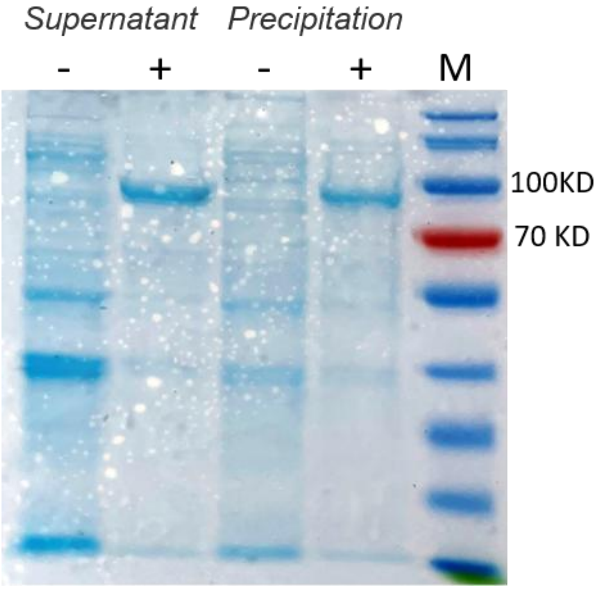

Figure S8. The SDS-PAGE analyzed the expression of GdpP protein. The *E. coli* strain BL21(DE3) carrying the pET28a-GdpP plasmid was incubated in LB medium at 37 ℃ with shaking (at 220 rpm) to logarithmic growth phase, and then induced expression at 25 ℃ for 14 h with or without 1 mM IPTG. The cells were lysed by sonication and centrifuged at 6000 x g for 30 minutes at 4°C to get the supernatant and precipitation for SDS-PAGE. +, IPTG; -, NO IPTG

Supplementary Table 1. qRT-PCR primers for detection genes and construction of the *gdpP* gene overexpressing strains

Primers	Sequences（5'-3'）
agrA-F	GCAGTAATTCAGTGTATGTTCA
agrA-R	TATGGCGATTGACGACAA
luxS-F	CGGACTACATTCATTAGAACATT
luxS-R	TTACAAGCAGGCACTTCA
sarA-F	CTTGTGGTTGTTTGTAGTTT
sarA-R	GTTATCAATGGTCACTTATGC
sigB-F	TGAGTGTCCATAAGAATCC
sigB-R	ATAACGATGGCACAATGA
saeR-F	GTCGTAACCATTAACTTCTG
saeR-R	ATCGTGGATGATGAACAA
atl-F	TATGGCTCTGTGAATGGTAA
atl-R	GGCTTAGGTGTTGGTGTA
cidA-F	TCATTCATAAGCGTCTACA
cidA-R	TCTTCATACCGTCAGTTG

clfA-F	GCTTCAGTGCTTGTAGGTA
clfA-R	GCTATCAGATTGCGTAACAC
fnbB-F	ACGACTGGTTGTTAGGTT
fnbB-R	GTGAGACTACGGTTAGCA
icaA-F	TCAGATAATACAGCAGAACTCA
icaA-R	GCATCCAAGCACATTACATAA
icaB-F	CCTATCCTTATGGCTTGATGA
icaB-R	CATTGGAGTTCGGAGTGA
cap5A-F	AATACGCAACTTATCAACA
cap5A-R	CACCGATTAGATTCACTAC
hla-F	CTCGTTCGTATATTACATCTAT
hla-R	GGTATATGGCAATCAACTT
hlg-F	GTGGCTCATTCAACTACTC
hlg-R	GCAGATACTTGACCATTCG
hld-F	CACTGTGTCGATAATCCATT
hld-R	AGGAAGGAGTGATTTCAATG
hlb-F	GCGTAGCGATTGTAAGTA
hlb-R	TCTTCAGATTGTGTATGTGTA
lukDE-F	GTCATAACTATCTCTACCAT
lukDE-R	TTACAGAACTACGATTGAT
lukpvl-S-F	CGCTACTTGTATCTTCTGTT
lukpvl-S-R	CATTGTCGTTAGGAATAATCAC
beta PSM-F	CGCAATTAAAGATACCGTAACT
beta PSM-R	TTCAACGATGCTCACAATG
delta PSM-F	CACTGTGTCGATAATCCATT
delta PSM-R	AGGAAGGAGTGATTTCAATG
gyrb-F	ACATTACAGCAGCGTATTAG
gyrb-R	CTCATAGTGATAGGAGTCTTCT
pCN51-gdpP-R BAMHI	CGCggatccTCATGCATCTTCACTCCTACTTAATTGTTC
pCN51-gdpP-F KPN1	CGGggtaccATGAATCGGCAGTCCACTAAGAAA
